# The Medical Year

**Published:** 1910-12-31

**Authors:** 


### The Medical Year.

The twelvemonth just closing has not been an epoch-making period so far as the advance of medicine is concerned. Looking at it with unprejudiced eyes, and reviewing calmly the events that have been worthy of special record, one is struck by the barrenness of the harvest in general medicine and surgery and by the absence of any particularly striking performances in the realm of specialisation. The year 1909 closed with brilliant promise of achievement, but the fulfilment has not realised the hopes held out by enthusiasts when 1910 was yet young. In medicine the advances in vaccine therapeutics and sero-therapy have been singularly slow; there are many who seriously incline to the belief that in this special department of general medicine retrogression and failure have been as marked as progress and success during the past twelve months. We have yet to summarise our conclusions with regard to another much-vaunted novelty—the apotheosis of the arsenical compounds in the advent of the now famous 606. It is possible that the high hopes entertained six months ago, when the limitations of this new remedy were not so well known as they are now, may prove as impossible of realisation as the brilliant promise of serum therapy and vaccine treatment. In the domain of surgery we have to chronicle nothing of especial noteworthiness in England. On the Continent and in America the year has been marked by an encouraging advance in the region of spinal and thoracic operations. The use of the Sauerbruck and Brauers cabinets has given anæsthetists an additional problem to solve—namely, the narcotisation of patients under low or high pressure—and the attempt to deal with this question in a practical manner has led to the development of special and complicated anæsthetising apparatus which are yet on their trial.

With regard to individual operations, that devised by Forster, of Breslau, for cases of tabetic gastric crises and cases of spastic diplegia has attracted considerable attention, and it is worth while, too, to notice the recent development of neuroplastic operations generally. Old methods, such as the hyperæmic treatment, tuberculin injections, radium and light treatment, have retained their popularity, and the progress made in therapeutics, so far as these newer auxiliaries are concerned, has on the whole been distinctly encouraging. On the other hand, we have failed to achieve anything which may honestly be styled great in the departments which have of late years been so actively studied by pioneers. There are no striking novelties to record in tropical medicine, in cancer research, in ophthalmology, in obstetrics, and allied departments. The fertile field of comparative medicine has been almost wholly neglected, and there has been no desire shown to renew the agitation on behalf of the establishment of a special school, at Cambridge or elsewhere, to be devoted to research in this domain. The supporters of the scheme for a central school for the investigation of tropical diseases have been at some pains to direct public attention to the project by suggesting that such a centre should be started as a national metropolitan memorial to the late King. This suggestion, apparently, has so far found no favour, but there is still a chance that it may receive the consideration its importance warrants.

A very gratifying development has been noticed during the past year in one special department—pediatrics. The problem of the school child, of the school clinic, and questions of medical inspection of schools generally, have all combined to interest the profession and the public as well. Such interest has undoubtedly tended to influence research and activity in this department, and, although we cannot point to any specially brilliant works or achievements done during 1910, it is yet an undoubted fact that the mass of observation, of isolated investigation and combined activity, has been productive of much good, and we may look forward to seeing even better work accomplished in this department in 1911.

